# Cost-effectiveness of abbreviated breast MRI and contrast-enhanced mammography for women with extremely dense breasts in a national breast cancer screening program: a micro-simulation study

**DOI:** 10.1016/j.breast.2026.104907

**Published:** 2026-08-31

**Authors:** Mengmeng Li, Marcel J.W. Greuter, Keris Poelhekken, Monique D. Dorrius, Karin M. Vermeulen, Antoinette D.I. van Asselt, Geertruida H. de Bock

**Affiliations:** a Department of Epidemiology, University Medical Center Groningen, University of Groningen, Groningen, the Netherlands; b Department of Radiology, University Medical Center Groningen, University of Groningen, Groningen, the Netherlands; c Department of Health Science, University Medical Center Groningen, University of Groningen, Groningen, the Netherlands

**Keywords:** Breast neoplasms, Mass screening, Breast density, Magnetic resonance imaging, Contrast-enhanced mammography, Cost-benefit analysis

## Abstract

**Background:**

Extremely dense breast tissue is associated with increased breast cancer risk and reduced sensitivity of digital mammography (DM). This study evaluated the cost-effectiveness of abbreviated breast MRI (AB-MRI) and contrast-enhanced mammography (CEM) versus DM for screening women with extremely dense breasts.

**Methods:**

This micro-simulation study applied the validated SimRisc Breast model. Screening strategies included biennial supplemental full-protocol MRI (FP-MRI), supplemental AB-MRI, supplemental CEM, AB-MRI alone and CEM alone, with biennial DM (ages 50-74) as reference. Costs and life-years gained (LYG) were discounted at 3%. Average cost-effectiveness ratios (ACERs) were calculated versus the reference, and incremental cost-effectiveness ratios (ICERs) for pairwise comparisons. A willingness-to-pay threshold of €20,000 per LYG was applied.

**Results:**

Compared with the reference, all alternative screening strategies increased cancer detection and LYG, while reducing interval cancers and breast cancer deaths. CEM alone and supplemental CEM slightly increased in radiation-induced tumors. In cost-effectiveness analysis, supplemental AB-MRI and supplemental FP-MRI were not favorable (ACERs of €24,700–€34,400 per LYG), whereas CEM alone, AB-MRI alone, and supplemental CEM were cost-effective (ACERs €13,400–€19,600 per LYG). Incremental comparisons indicated that all supplemental strategies were dominated, with CEM alone optimal (ICER €13,400 per LYG vs. reference) and AB-MRI alone at €49,400 per LYG relative to CEM alone.

**Conclusion:**

Both AB-MRI and CEM could reduce breast cancer mortality through earlier detection, with low additional risks and reasonable incremental costs. Among the modeled strategies, CEM was estimated to be the most cost-effective, although further prospective clinical studies are needed to confirm these findings.

## Highlights

• The micro-simulation model (Simrisc Breast) was validated using the DENSE trial data.
• AB-MRI and CEM lower breast cancer deaths vs. DM with acceptable risks and costs.
• AB-MRI and CEM are cost-effective alternatives to DM for extremely dense breasts.
• CEM alone is the optimal screening strategy for extremely dense breasts.
• CEM's cost-effectiveness is most influenced by its screening sensitivity and cost.

## Graphical abstract

**Figure:**
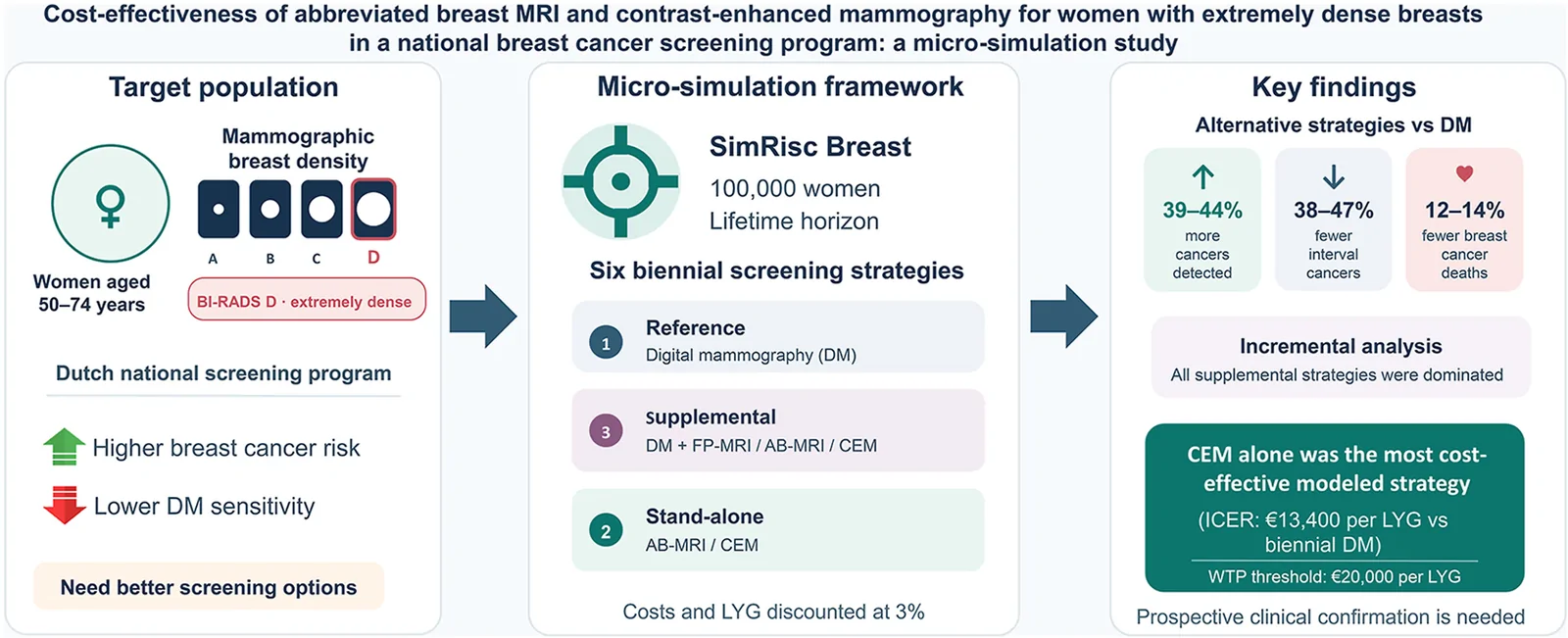

## 1 Introduction

Breast cancer is the most common cancer among women in the Netherlands, with a lifetime risk of approximately 1 in 7 [1]. To reduce mortality, national mammography screening programs have been implemented in most developed countries, leading to an average 23% reduction in breast cancer deaths [2]. However, the sensitivity of biennial digital mammography (DM) is limited in women with extremely dense breasts, who comprise about 8% of the screening population [3], decreasing from 85.7% in women with almost entirely fatty breasts to only 61% in this subgroup [4]. Moreover, having extremely dense breast tissue is associated with an increased likelihood of developing breast cancer, which is about twice as high as in average-risk women and 4-6 times higher than in women with almost entirely fatty breasts [3].

Given the combined challenge of lower detection rates and increased cancer risk in this group, there is a clear need to explore potential supplemental or alternative imaging modalities to DM to improve screening effectiveness. Based on findings from the Dense Tissue and Early Breast Neoplasm Screening (DENSE) trial [5,6] and cost-effectiveness evidence presented by Geuzinge et al. [7], the European Society of Breast Imaging (EUSOBI) recommended in 2022 that supplemental full protocol MRI (FP-MRI) screening should be offered to women aged 50-70 with extremely dense breasts at least every 4 years, and preferably every 2 to 3 years [3]. However, practical barriers such as high costs and limited FP-MRI availability make widespread implementation of FP-MRI screening for the general population unlikely [8].

To address these barriers, two alternative imaging modalities have gained increasing attention. Abbreviated breast MRI (AB-MRI) shortens acquisition and interpretation time, while maintaining diagnostic performance comparable to FP-MRI [9,10]. Previous modeling studies suggest that AB-MRI may be cost-effective for women with dense breasts [11,12]. More recently, contrast-enhanced mammography (CEM) has emerged as another potential alternative, offering faster image acquisition and lower costs than FP-MRI [13]. CEM combines standard DM with an iodine-based contrast agent to generate images highlighting vascular enhancement and has shown comparable diagnostic performance to FP-MRI [14]. Although primarily used in diagnostic settings [15], recent studies suggest potential for screening applications [16,17]. For example, when used as a screening tool among women with extremely dense breast tissue at intermediate risk, CEM showed significantly higher sensitivity compared to standard DM in a retrospective study of 609 women, 88.9% vs 27.8% [18].

To investigate the potential of AB-MRI and CEM for early detection of breast cancer in women with extremely dense breasts, a large population-based trial (DENSE-2) was launched in late 2024. However, it will take several years before robust evidence on the effectiveness of these modalities becomes available. In addition, it remains unclear which modality - AB-MRI or CEM – is more cost-effective for women with extremely dense breasts in population-based screening. Micro-simulation studies therefore provide an appropriate approach to evaluate the long-term benefits, harms, and cost-effectiveness of these screening strategies. Using a micro-simulation model, we aimed to assess whether AB-MRI and CEM could serve as cost-effective options for women with extremely dense breasts within the Dutch national breast cancer screening program.

## 2 Methods

This micro-simulation study was reported according to the Consolidated Health Economic Evaluation Reporting Standards (CHEERS) statement [19]. The previously validated micro-simulation model SimRisc Breast was applied in this analysis [[20], [21], [22]].

### 2.1 Model summary

In brief, women were simulated individually over their lifetime, accounting for life expectancy, breast cancer incidence, tumor growth, tumor self-detection probability and survival probability. Only invasive breast cancers were modeled. If no tumor developed and death occurred from causes other than breast cancer, survival was determined using age-specific mortality in the general population. For women who developed a tumor, detection through screening or self-detection was determined by the sensitivity of the screening modality or the probability of self-detection. Following breast cancer diagnosis, women exited the simulation, and breast cancer-specific mortality was estimated based on post-diagnosis life expectancy, which was influenced by tumor size. The model also incorporated false positives and estimated the risk of radiation-induced tumors when ionizing radiation was used. A detailed description of the model is available in previous publications [11,[20], [21], [22]].

### 2.2 Input parameters

Model input parameters, as displayed in Table 1 were derived from population statistics and systematic literature searches. To adapt the SimRisc Breast model for screening women with extremely dense breasts, we updated relevant parameters (illustrated below) from Wang et al. using data specific to this population [11].

**Table 1:** Input variables and their estimates for the Simrisc Breast model.

	Variables		Base value (95% CI)				Reference
Tumor incidence model	Lifetime risk [%]		24.7% (23.4-25.9)				[1,2]
	Mean onset age [years]		67.6 (66.2-68.9)				
	Spread [years]		17.1 (16.1-18.2)				
Tumor growth model	Tumor volume doubling time (/age group)	< 50 years	80 (44-147) days				[3]
		50-70 years	157 (121-204) days				
		>70 years	188 (120-295) days				
Self-detection diameter [mm]	Mean of self-detection size		18.541				[4]
	Spread of self-detection size		2.014				
Mammographic breast density	BI-RADS density distribution		a	b	c	d	[5]
	50-60 years		0	0	0	1	
	60-70 years		0	0	0.69	0.31	
	>70 years		0	0.15	0.62	0.23	
Participation	Participation rate		100%				[6]
Tumor induction model	Excess relative risk of tumor induction due to radiation per Gy		0.51 (0.28-0.83)				[7]
	Radiation dose (per screen) in mGy	BI-RADS density	a	b	c	d	[8]
		Mammography	4.01 (3.22-5.05)	4.01 (3.22-5.05)	4.22 (3.24-5.33)	2.70 (2.06-4.61)	
		CEM	5.90 (5.03-6.83)	5.90 (5.03-6.83)	6.02 (4.47-7.52)	4.52 (3.06-6.98)	
DM	Sensitivity beta function	Mean area percent density (m)	0.06 (0.02-0.1)	0.16 (0.08-0.25)	0.40 (0.19-0.61)	0.83 (0.65-1.00)	[9]
	Specificity [%]		95.5 (95.0-96.1)				[10]
	Costs/screen		81				[11,12]
	Systematic error detection		10%				[13]
FP-MRI	Sensitivity [%]		95.2 (88.1-98.7)				[14]
	Specificity [%]		92.0 (91.2-92.8)				
	Costs/screen		334				[12,15]
AB-MRI	Sensitivity [%]		95.7 (79.0-99.2)				[16]
	Specificity [%]		86.7 (84.8-88.4)				
	Costs/screen		223				[12,15,17]
CEM	Sensitivity [%]		85.7 (42.1-99.6)				[18]
	Specificity [%]		85.9 (82.4-88.9)				
	Costs/screen		162				-
Costs in case of positive finding	Biopsy		234				[12,19]
	Treatment (tumor diameter)	< 2 cm	8436				[12,20]
		2-5 cm	9340				
		> 5 cm	10091				

Abbreviations: CI = confidence interval; BI-RADS = Breast Imaging Reporting and Data System; DM = digital mammography; FP-MRI = full-protocol MRI; AB-MRI = abbreviated breast MRI; CEM = contrast-enhanced mammography.

#### 2.2.1 Tumor incidence model

Age-specific incidence rates of invasive breast cancer in the general population were estimated based on data from the Netherlands Cancer Registry (NCR), managed by the Netherlands Comprehensive Cancer Organisation (IKNL) [23]. Then, these rates were adjusted for women with extremely dense breasts based on the reported relative risks from Schousboe et al. [24]. Details can be found in Supplementary Table S1 and Table S2. Among women with extremely dense breasts, the estimated lifetime risk was 24.7%, and the mean tumor onset age was 67.6 years - 3.1 years earlier than the 70.7 years observed in the general population (Supplementary Fig. S1).

#### 2.2.2 Mammographic breast density

Based on Dutch data [25], we assumed that breast density decreases with age. 23% of the women with category d density at age 50 were modeled to remain at that level. For 77% of the women, a decline in breast density was modeled, at the age of 60 and/or 70 years (Supplementary Table S3). The distribution of breast density across different age group is shown in Table 1.

#### 2.2.3 Radiation dose

The estimates for radiation dose were derived from Sanders et al. [26], which reported median mean glandular doses (MGD) for two-review full-field DM and CEM in women undergoing breast cancer screening. For DM, median MGDs were 4.01, 4.22, and 2.70 mGy for breast density categories b, c, and d, respectively; for CEM, the corresponding values were 5.90, 6.02, and 4.52 mGy. Because no data were available for BI-RADS a, we applied the MGD for category b in the model: 4.01 mGy for DM and 5.90 mGy for CEM.

#### 2.2.4 Test performance of DM, FP-MRI, AB-MRI and CEM

Among women with extremely dense breasts, DM demonstrated a specificity of 95.5% [27]. The sensitivity of AB-MRI was comparable to that of FP-MRI, 95.7% and 95.2%, respectively, while the specificity of AB-MRI was lower than that of FP-MRI, 86.7% versus 92.0% [5,9]. For CEM, the sensitivity and specificity were 85.7% and 85.9%, respectively [18].

### 2.3 Costs

The costs considered in this study included screening tests (DM, FP-MRI, AB-MRI and CEM), biopsies, and breast cancer treatment according to tumor size at diagnosis (Table 1). The cost of DM per screen was €81, which was derived from the Dutch national screening program report [28]. The cost for AB-MRI was estimated at two-thirds of the FP-MRI cost, i.e. €223 per screen compared with €334 for FP-MRI [29], based on higher throughput of AB-MRI, where three to four women can be screened per hour compared with two using full-protocol MRI [30]. The cost per CEM screen was set at €162 based on breast imaging expert opinion (MDD), reflecting the additional resources required for CEM, including the use of iodinated contrast material, IV line placement, extra technologist time, and the need for monitoring during contrast administration [13]. All costs were indexed to 2024 values according to the Dutch consumer price index [31].

### 2.4 Screening strategies

**Fig. 1:**
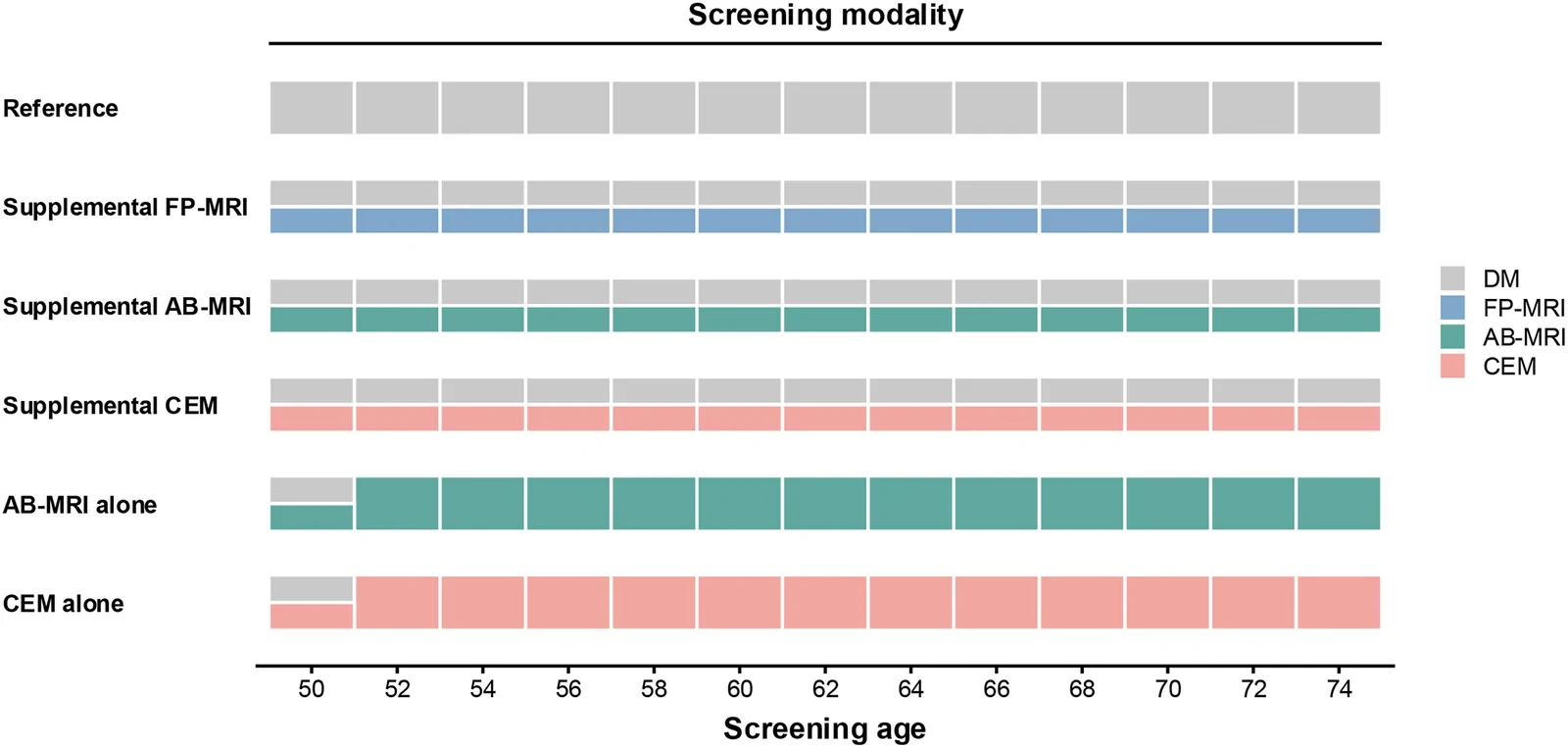
All screening strategies visualized. Abbreviations: DM = digital mammography; FP-MRI = full-protocol MRI; AB-MRI = abbreviated breast MRI; CEM = contrast-enhanced mammography.

Six screening strategies were simulated (Fig. 1). The current Dutch population-based breast cancer screening strategy, biennial DM for women aged 50–74 years, served as the reference strategy. The supplemental FP-MRI strategy used in DENSE trial was also modeled. Four additional strategies were evaluated incorporating AB-MRI and CEM either as supplemental or alternative modalities to DM for women with extremely dense breasts: supplemental AB-MRI, supplemental CEM, AB-MRI alone and CEM alone. In all strategies, screening began with DM at age 50, since women always undergo DM at their initial screening (due to unknown density). If breast density decreased from category d to c, screening switched to biennial DM. We assumed that breast density can be measured with DM, FP-MRI, AB-MRI and CEM. In the supplemental screening strategy, women first undergo DM, allowing cancellation of the FP-MRI, AB-MRI or CEM if DM indicates decreased density. A 100% screening participation rate was modeled in the base-case analysis to enable standardized comparison of screening strategies under equal uptake, consistent with established breast cancer screening micro-simulation studies [7,32].

### 2.5 Outcomes and cost-effectiveness

We simulated 100,000 women to minimize statistical error and limit computation time and repeated each simulation 10 times. Model outcomes included screen-detected cancers, radiation-induced tumors, breast cancer deaths, interval cancers and life years gained (LYG) per 100,000 women over their lifetimes. The average cost-effectiveness ratios (ACERs) were estimated as the ratios of the additional costs of each screening strategy to the LYG compared with the reference (Dutch strategy). In addition, incremental cost-effectiveness ratios (ICERs) were calculated by comparing the less expensive strategy with the next more costly and effective strategy after excluding dominated strategies. A discount rate of 3% for both costs and health effects (LYG) was applied to allow for international comparisons [33]. As no formal Dutch willingness-to-pay threshold has been established for LYG, we used €20,000 per LYG as a reference threshold, informed by the Dutch reference threshold of €20,000 per quality-adjusted life-year (QALY) [34].

### 2.6 Sensitivity analysis

To identify the individual model parameters that had the greatest influence on the cost-effectiveness results, a univariate sensitivity analysis was conducted for the optimal screening strategy identified in the base-case cost-effectiveness analysis. ICERs were estimated by comparing this optimal screening strategy with the reference strategy. Each input parameter in Table 1 was adjusted one by one to the minimum and maximum estimates of its 95% confidence interval (CI). In addition, uncertainty due to the screening test cost was assessed by varying the cost by ±25%. The screening participation rate was varied from 100% in the base-case analysis to the current Dutch level of 65.3% [35] to assess its influence on the ICER under routine screening conditions. In this analysis, each woman had a 65.3% probability of attending screening at a given invitation round. The impact of parameter uncertainty on ICERs was illustrated using tornado plots.

If the CEM-based screening strategy was identified as the optimal strategy in the base-case analysis, two additional analyses were performed. First, a scenario analysis was conducted using the recently published Dutch estimate of €111 per examination [36] instead of the base-case cost of €162. Second, a threshold analysis was performed by progressively reducing CEM sensitivity from the base-case estimate (85.7%) to determine the threshold at which the ICER exceeded the willingness-to-pay threshold.

### 2.7 Validation of the model

The population used for model validation consisted of Dutch women aged 50-75 years with extremely dense breasts, similar to those in the DENSE trial. Simulated outcomes included the number of screen-detected cancers and false positives during the first (prevalent) and second (incident) screening rounds, as well as interval cancers during the first round, in both the reference and supplemental FP-MRI scenarios. The 95% CI of the simulated outcomes were derived through univariate sensitivity analysis and was compared with 95% Poisson confidence intervals of the observed numbers from the DENSE trial for the control and MRI participants group. The simulated results were considered valid when the 95% CIs of the observed data and simulated outcomes overlapped.

## 3 Results

### 3.1 Validation of the model

Supplementary Table S4 shows the numbers of observed and simulated interval cancers, screen-detected cancers and false positives. Most of the 95% CIs of the simulated results overlapped with 95% CIs of the observed data. For the mammography-only group, the number of T1 interval cancers in the first round was overestimated, as well as the number of T1 screen-detected cancers in the second round, while the number of T2+ interval cancers in the first round was underestimated. For the MRI participants group, the number of T2+ and total screen-detected cancers in the first round were underestimated, whereas the number of false positives in the second round by MRI in the MRI participants was overestimated.

### 3.2 Estimated benefits, harms and cost-effectiveness of all screening strategies

The estimated benefits, harms and cost-effectiveness of all screening strategies are presented in Table 2, ordered from lowest to highest total costs. Compared with the reference, the use of CEM alone, AB-MRI alone, supplemental CEM, supplemental AB-MRI and supplemental FP-MRI reduced breast cancer deaths by 12%-14%, detected 39%-44% more cancers, and decreased interval cancers by 38%-47%. In addition, the use of AB-MRI alone reduced radiation-induced tumors by 60%. These screening strategies also yielded more life years, with discounted LYG ranging from 456 to 522 per 10,000 women compared to DM alone screening. Regarding harms, both CEM alone and supplemental CEM resulted in a slight increase in radiation-induced tumors from 0.2% to 0.31% and 0.39% respectively.

**Table 2:** Benefits, harms and cost-effectiveness of all screening strategies per 10,000 screened women.

Screening strategies	Screen-detected cancers	Radiation-induced tumors	Cancer deaths	Interval cancers	No. of mammograms	No. of CEMs or MRIs	Discounted additional cost^a^ (k€)	Discounted LYG^a^	Discounted ACER^a^ (k€/LYG)	Discounted ICER^a^ (k€/LYG)
Reference	574	20	500	508	96462	-		-	-	-
CEM alone	799	31	442	307	41702	63009	6199	462	13.4	13.4
Supplemental CEM	798	39	444	313	96211	54839	8951	456	19.6	ED
AB-MRI alone	827	8	429	270	41735	63012	9161	522	17.6	49.4
Supplemental AB-MRI	814	20	435	289	96248	54856	11562	468	24.7	ED
Supplemental FP-MRI	813	20	436	289	96248	54856	16033	467	34.4	ED

Abbreviations: LYG = life years gained; ACER = Average cost-effectiveness ratio; ICER = incremental cost-effectiveness ratio; FP-MRI = full-protocol MRI; AB-MRI = abbreviated breast MRI; CEM = contrast-enhanced mammography; ED = Extended dominance.

a A discounting rate of 3% was applied for both costs and LYG.

When applying a 3% discount rate to both LYG and costs, we found that supplemental AB-MRI and supplemental FP-MRI were not cost-effective compared with the reference strategy, with ACERs of €24,700 to €34,400 per LYG. In contrast, CEM alone, AB-MRI alone and supplemental CEM screening strategies had ACERs below the willingness-to-pay threshold, ranging from €13,400 to €19,600 per LYG, indicating that these strategies may be considered cost-effective relative to DM alone screening. However, incremental cost-effectiveness analysis comparing all strategies showed that all supplemental strategies were dominated. Among the stand-alone strategies, AB-MRI alone had an ICER of €49,400 per LYG, whereas CEM alone had an ICER of €13,400 per LYG. Based on incremental comparison and a willingness-to-pay threshold of €20,000 per LYG, CEM alone screening appeared to be the optimal strategy among the modeled screening scenarios.

### 3.3 Sensitivity analyses

**Fig. 2:**
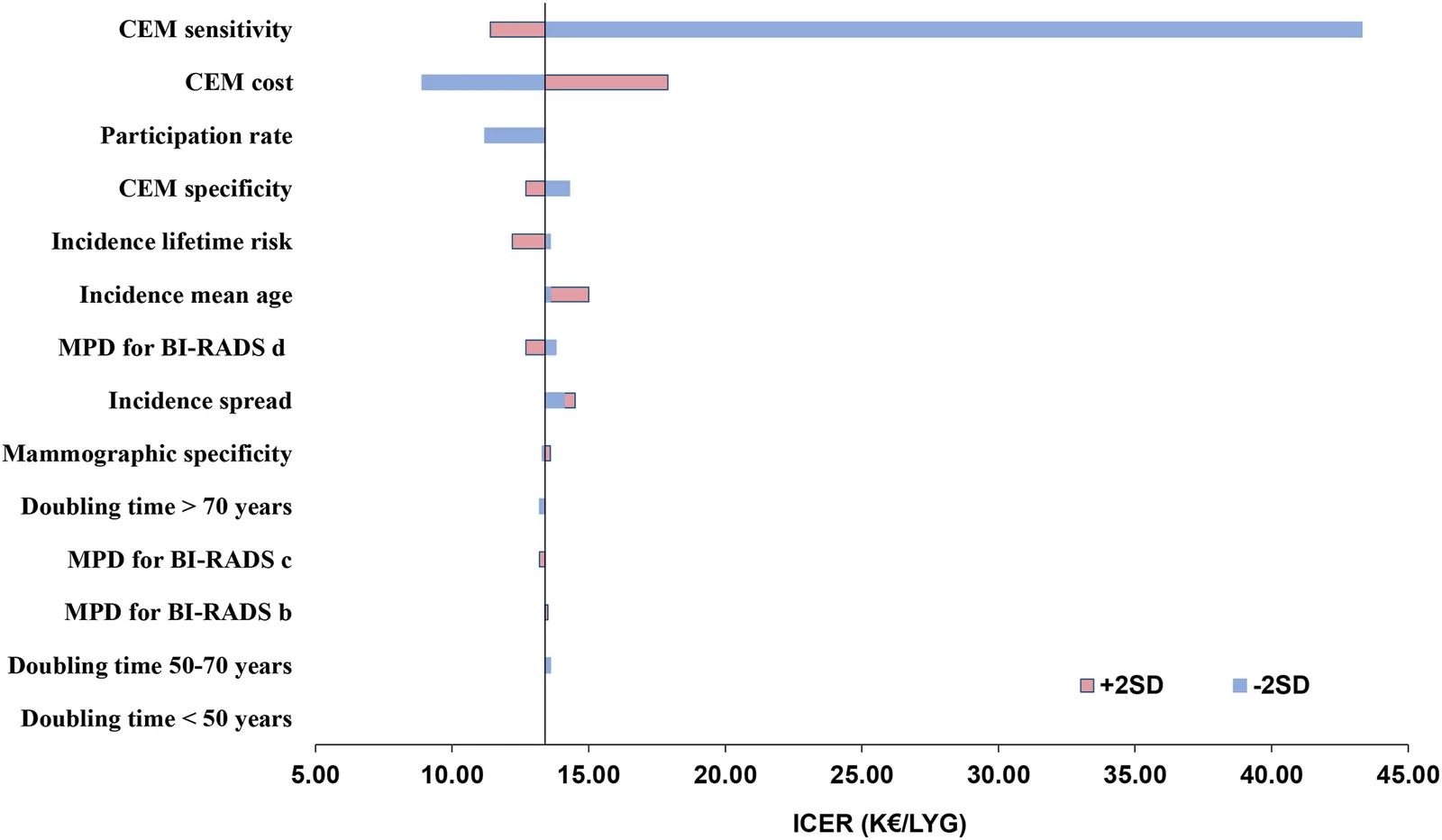
Tornado plot of the univariate sensitivity analysis for the CEM alone screening strategy vs the reference. Abbreviations: CEM = contrast-enhanced mammography; MPD = mean percent density; BI-RADS = Breast Imaging Reporting and Data System; SD = standard deviation; LYG = life year gained; ICER = Incremental cost-effectiveness ratio.

A summary of the univariate sensitivity analysis for the CEM alone screening strategy is shown in Fig. 2. The ICER of CEM alone screening was most affected by the sensitivity of CEM, with values ranging from €11,400 to €43,300 per LYG. Fig. S2 shows that CEM remained cost-effective as long as the sensitivity of CEM did not decrease below 63.7%. The cost of CEM per screen also had a substantial influence, with values ranging from €8900 to €17,900 per LYG. In the scenario analysis using the recently published Dutch estimate of €111 per CEM examination, the ICER of the CEM-alone strategy decreased from €13,400/LYG in the base-case analysis to €7800/LYG (Table S5). Other parameters, including participation rate, CEM specificity, tumor incidence parameters, and MPD for BI-RADS d had a moderate impact on the ICER. In contrast, uncertainties in mammography specificity, tumor doubling time and MPD for BI-RADS b and c had only a minor impact on the ICER.

## 4 Discussion

In this micro-simulation study, we assessed the cost-effectiveness of AB-MRI and CEM for women with extremely dense breasts in a population-based breast cancer screening program. Using a micro-simulation model, validated by accurately reproducing the observed data of the first and second screening rounds of the DENSE trial, we found that compared to the reference, biennial DM screening from age 50-74, biennial AB-MRI alone, CEM alone and supplemental CEM screening were all cost-effective, with ACERs ranging from €13,400 to €19,600 per LYG. In addition, our model identified that all supplemental screening strategies were dominated and biennial CEM alone screening was optimal with highest acceptable ICER of €13,400 per LYG. The univariate sensitivity analysis showed the ICER of CEM alone screening was most strongly influenced by the sensitivity of CEM, followed by the cost of CEM per screen.

Given that women with extremely dense breasts face both an increased risk of breast cancer and substantially reduced DM sensitivity, supplemental and alternative screening strategies have been increasingly explored. The multicenter Dutch DENSE trial demonstrated that supplemental FP-MRI screening substantially reduced interval cancer rate (2.5 per 1000 screenings vs 5.0 per 1000 screenings in the mammography-only group) [5]. To extrapolate these findings and assess long-term outcomes, Geuzinge et al. applied an updated MISCAN micro-simulation model to evaluate the cost-effectiveness of several FP-MRI screening strategies, reporting that adding biennial FP-MRI to biennial DM, as implemented in the DENSE trial, would cost approximately €22,532 per quality-adjusted life-year (QALY) [7]. In line with the modeling results of Geuzinge et al., our analysis showed that the DENSE trial screening strategy (biennial supplemental FP-MRI) was also not cost-effective compared with biennial DM, with an ACER of €34,400 per LYG. The modeling work by Geuzinge et al. also suggested that FP-MRI used as a stand-alone screening modality, rather than as a supplement to DM, may provide a more favorable balance between costs and health benefits, with a four-year screening interval emerging as the most efficient strategy [7]. Nevertheless, the high costs and limited availability of FP-MRI pose substantial barriers to its widespread implementation, underscoring the need for more affordable and broadly applicable alternative approaches.

Both AB-MRI and CEM have emerged as promising, lower-cost alternatives to FP-MRI, offering substantially shorter acquisition and interpretation times, while maintaining high diagnostic accuracy [37]. Our results showed that AB-MRI alone is cost-effective for screening women with extremely dense breasts compared with DM, with an ACER of €17,600 per LYG. Several previous studies have also evaluated the cost-effectiveness of AB-MRI in women with dense breast tissue, and consistent findings were reported. Wang et al. reported that biennial AB-MRI screening between the ages of 50-65 for only women with extremely dense breasts could be a cost-effective alternative to mammography, yielding an ACER of approximately €16,000 per LYG [11]. Similarly, Tollens et al. showed that AB-MRI may be considered cost-effective compared with FP-MRI for breast cancer screening in women with dense breasts, provided that the cost per examination does not exceed 82% of that of FP-MRI [12].

To date, no studies have specifically assessed the cost-effectiveness of CEM as a population-based screening tool. Existing evidence is limited to its use as a primary diagnostic modality, where CEM has been shown to be cost-effective [38]. Our study extends this evidence by demonstrating that CEM may also be a cost-effective screening option for women with extremely dense breasts, both as a supplemental strategy and as a stand-alone modality. Although supplemental CEM is cost-effective and is currently being evaluated in the DENSE-2 trial, it should be noted that CEM inherently includes a mammographic acquisition; therefore, women undergoing CEM would not receive an additional DM. Consequently, in clinical and screening practice, CEM is intended to be implemented as a stand-alone screening modality rather than in combination with DM.

CEM appears to be more cost-effective than AB-MRI. Our results showed that the ICER for AB-MRI alone relative to CEM alone was €49,400 per LYG, well above the €20,000 willingness-to-pay threshold. The more favorable cost-effectiveness of CEM is likely driven by several practical advantages. AB-MRI and CEM show comparable sensitivity and specificity for breast cancer screening, as confirmed by the BRAID trial, in which CEM even demonstrated slightly higher cancer detection than AB-MRI [39]. In addition to its high diagnostic performance, CEM is less expensive than MRI and can be integrated into conventional DM or tomosynthesis systems, thereby lowering barriers to adoption and enhancing patient access [40]. Moreover, unlike MRI, CEM can be safely performed in patients with claustrophobia or those with metallic implants [41], allowing for easier scheduling and broader applicability. However, the use of CEM as a screening modality also has several disadvantages. First, CEM still requires prolonged breast compression and is subject to multiple contraindications, including breast implants, renal or heart failure, diabetes mellitus, and allergy to iodine-based contrast agents. Second, because of the risk of contrast-related allergic reactions, including anaphylactic shock, CEM must be performed in a hospital setting rather than in mobile breast cancer screening units.

The ICER of the CEM alone screening strategy was most sensitive to changes in the sensitivity of CEM. In the univariate sensitivity analysis, reducing CEM sensitivity to the lower bound of its 95% confidence interval increased the ICER to €43,300/LYG, exceeding the willingness-to-pay threshold of €20,000/LYG. The threshold analysis further showed that CEM-alone screening remained cost-effective when CEM sensitivity was above 63.7%; below this value, the ICER exceeded the willingness-to-pay threshold. These findings indicate that the cost-effectiveness of CEM-alone screening depends strongly on the assumed CEM sensitivity. Although the source estimate was imprecise, larger CEM screening studies have reported sensitivity estimates substantially above the identified threshold of 63.7%. For example, Sorin et al. recently reported 5424 CEM screening examinations in women at intermediate to high risk; 84.9% had dense breasts, and CEM achieved a sensitivity of 95.9% (71/74) [16]. Similarly, Patel et al. reported prevalence-round results from a prospective study of supplemental screening with CEM in women at elevated risk. Among 460 participants, 88.7% had mammographically dense breasts, and CEM demonstrated high sensitivity (91.7%; 95% CI, 0.760–1.000) [17]. Although these findings provide some reassurance that CEM sensitivity may remain above 63.7%, differences in study populations and designs limit direct comparison. The univariate sensitivity analysis also showed that the cost of CEM per screening examination had a substantial impact on the ICER, which is consistent with previous studies [7,11,42]. Nevertheless, uncertainty in this parameter did not affect the overall conclusion, as all ICER estimates remained below the willingness-to-pay threshold. Similarly, although the participation rate had a moderate impact on the ICER, applying the current Dutch participation rate of 65.3% did not alter the cost-effectiveness conclusion, as CEM-alone screening remained below the willingness-to-pay threshold.

In this study, we did not include digital breast tomosynthesis (DBT) in the analysis. While systematic reviews and meta-analyses report higher pooled sensitivity for DBT than for DM (91% vs. 86%) [43], recent evidence shows that its sensitivity drops with increasing breast density, reaching only 61.8% in women with extremely dense breasts [44]. Therefore, we do not expect this population to gain substantial benefit from DBT, and we did not consider it as a potential screening strategy.

This study has several strengths. First, we utilized data from both incident and prevalent screening rounds of a large, randomized controlled trial to validate the SimRisc Breast model [5,6], ensuring the reliability of our findings. Second, instead of assuming that breast density remained unchanged [45] or continuing the use of supplemental or alternative modalities to mammography when density decreased [11], our model dynamically incorporates breast density distributions across different age groups. When density decreases from category d to c, our model switches to biennial DM. This approach allows our model to more accurately estimate the cost-effectiveness of the proposed screening strategies. Third, we directly compared multiple supplemental and alternative screening modalities to DM, including FP-MRI, AB-MRI, and CEM. Unlike other studies that focus on a single supplemental or alternative modality [7,11], our research provides a broader comparison, offering valuable insights for clinical decision-making.

Our study also has some limitations. First, although the simulated results generally showed no significant deviations from the observed data, the number of screen-detected cancers was underestimated in the MRI group during the first round. This may be attributable to the prevalence round effect observed in the DENSE trial and other prospective screening studies [6,46], where a larger pool of prevalent breast cancers is expected in the first screening round. The validation results also showed that the number of false positives was overestimated in the second round. This overestimation may arise from using a constant specificity derived from the first round of screening, whereas specificity typically improves in follow-up rounds. For example, in the DENSE trial, baseline specificity was 92%, while specificity in the second round increased to 97% [5,6]. Extending our model to incorporate round-specific specificity values would likely provide a more accurate estimation of false positives in breast cancer screening. Second, there is currently a lack of mature, standardized large-scale data directly comparing the performance of CEM with AB-MRI in women with extremely dense breasts. As a result, the diagnostic performance parameters for AB-MRI and CEM used in our model were derived from different published studies [9,18]. Although several initial studies suggest that CEM has a similar sensitivity but higher specificity than MRI [47,48], the specificity of CEM in our model was slightly lower than that of AB-MRI. We do not expect this limitation to substantially affect the conclusions of our study, as a higher specificity of CEM may make it an even more favorable strategy. Third, the reliability of micro-simulation results depends on the availability and quality of the input data. This is particularly relevant to CEM diagnostic performance, which was derived from a small, retrospective, single-center study in a selected higher-risk population [18]. The limited number of cancers detected in the baseline screening round resulted in a wide 95% confidence interval for CEM sensitivity (42.1%–99.6%). Both the univariate sensitivity and threshold analyses showed that the cost-effectiveness conclusion was sensitive to this parameter. Consequently, large-scale prospective studies focusing exclusively on women with extremely dense breasts who participate in national screening programs are required to obtain more robust and representative estimates of CEM diagnostic accuracy. Fourth, effectiveness was measured as LYG rather than QALY. The current version of SimRisc Breast model did not provide health-related quality of life or disutility associated with screening, false-positive findings, biopsies, contrast administration, or treatment. As QALY is relevant from a health economics perspective, future versions of SimRisc Breast may target to facilitate the quantification of QALY. Fifth, the model did not incorporate adverse events related to iodinated contrast administration or account for women who may not be eligible for CEM because of contraindications to contrast use. Although severe contrast reactions are rare, their exclusion may underestimate potential harms and costs associated with CEM screening. Finally, SimRisc Breast models only invasive breast cancer and does not include DCIS, which accounts for nearly 14% of screen-detected cancers in the Dutch population [35]. Therefore, the model could not estimate the potential effects of DCIS detection on overdiagnosis, overtreatment, costs, or health outcomes. This limitation is relevant because imaging modalities may differ in their detection of DCIS, and the direction and magnitude of its effects on effectiveness and cost-effectiveness remain uncertain. Future research should incorporate DCIS into the model to better evaluate its implications for screening outcomes.

## 5 Conclusion

AB-MRI and CEM may represent promising alternative screening strategies for women with extremely dense breasts within population-based breast cancer screening programs. In this micro-simulation model, both modalities were associated with improved cancer detection and reduced breast cancer mortality compared with DM alone. However, the findings remain dependent on model assumptions and currently available diagnostic performance data, particularly for CEM. Therefore, these results should be interpreted cautiously and require confirmation in large prospective clinical studies before broad implementation in routine screening practice.

## CRediT authorship contribution statement

**Mengmeng Li:** Writing – review & editing, Writing – original draft, Visualization, Validation, Software, Resources, Project administration, Methodology, Investigation, Formal analysis, Data curation, Conceptualization. **Marcel J.W. Greuter:** Writing – review & editing, Writing – original draft, Visualization, Validation, Supervision, Software, Resources, Project administration, Methodology, Investigation, Formal analysis, Data curation, Conceptualization. **Keris Poelhekken:** Writing – review & editing, Writing – original draft, Visualization, Validation, Software, Resources, Methodology, Investigation, Formal analysis, Data curation. **Monique D. Dorrius:** Writing – review & editing, Writing – original draft, Visualization, Validation, Supervision, Resources, Methodology, Investigation, Formal analysis. **Karin M. Vermeulen:** Writing – review & editing, Resources, Investigation. **Antoinette D.I. van Asselt:** Writing – review & editing. **Geertruida H. de Bock:** Writing – review & editing, Writing – original draft, Visualization, Validation, Supervision, Software, Resources, Project administration, Methodology, Investigation, Formal analysis, Data curation, Conceptualization.

## Ethical approval

Ethical approval was not required.

## Funding

This work was supported by 10.13039/501100004543China Scholarship Council (CSC) through a PhD scholarship awarded to ML (grant No. 202306100039).

## Declaration of competing interest

The authors declare the following financial interests/personal relationships which may be considered as potential competing interestsMengmeng Li reports financial support was provided by CSC (CSC No. 202306100039).

Given her role as [Editorial Board], [Geertruida de Bock] had no involvement in the peer review of this article and had no access to information regarding its peer review. Full responsibility for the editorial process for this article was delegated to another journal editor.
