# Supplementary material for: Cost-effectiveness of abbreviated breast MRI and contrast-enhanced mammography for women with extremely dense breasts in a national breast cancer screening program: a micro-simulation study
Source: Breast. 2026 Aug 31;90:104907. doi: 10.1016/j.breast.2026.104907 (PMC13563584; doi:10.1016/j.breast.2026.104907)
Supplement: Supplementary file 1 [file mmc1.docx]

**Breast cancer incidence in women with extremely dense breasts**

The age-specific incidence of new invasive breast cancer in the general population was obtained from the Netherlands Cancer Registry (1), using the most recent data from 2022. Relative risks of breast cancer for women with extremely dense breasts compared with the general population were derived from Schousboe et al (Table S1) (2). Applying these relative risks, we estimated the incidence of invasive breast cancer among women with extremely dense breasts. The estimated invasive breast cancer incidence used in the model is presented in the Table S2.

**Table S1.** Relative risk of breast cancer based on age and breast density (the reference group for each relative risk is all women of that age group) ^a^.

| BI-RADS ^b^ category | 40-49 years | 50-59 years | 60-64 years | 65-69 years | 70-79 years |
| --- | --- | --- | --- | --- | --- |
| a | 0.351 | 0.388 | 0.400 | 0.581 | 0.600 |
| b | 0.730 | 0.807 | 0.832 | 0.885 | 0.914 |
| c | 1.131 | 1.251 | 1.291 | 1.228 | 1.268 |
| d | 1.468 | 1.623 | 1.675 | 1.283 | 1.325 |

^a^ From Schousboe et al (2).

^b^ BI-RADS (American College of Radiology Breast Imaging Reporting and Data System) a= almost entirely fat; b = scattered fibroglandular densities; c = heterogeneously dense; d= extremely dense.

**Table S2.** Age dependent invasive breast cancer incidence rates.

| Age at diagnosis | General population incidence rate ^a^ (1) | Relative risk (2) | Estimated incidence rate in women with extremely dense breasts ^a^ |
| --- | --- | --- | --- |
| 0-4 | 0 | 1.468 | 0 |
| 5-9 | 0 | 1.468 | 0 |
| 10-14 | 0 | 1.468 | 0 |
| 15-19 | 0 | 1.468 | 0 |
| 20-24 | 2.65 | 1.468 | 3.89 |
| 25-29 | 13.14 | 1.468 | 19.29 |
| 30-34 | 44.26 | 1.468 | 64.97 |
| 35-39 | 94.69 | 1.468 | 139.00 |
| 40-44 | 146.72 | 1.468 | 215.38 |
| 45-49 | 225.60 | 1.468 | 331.17 |
| 50-54 | 279.31 | 1.623 | 453.32 |
| 55-59 | 256.15 | 1.623 | 415.73 |
| 60-64 | 324.42 | 1.675 | 543.40 |
| 65-69 | 389.85 | 1.283 | 500.18 |
| 70-74 | 462.80 | 1.325 | 613.21 |
| 75-79 | 345.93 | 1.325 | 458.36 |
| 80-84 | 338.70 | 1.325 | 448.36 |
| 85+ | 338.50 | 1.325 | 448.49 |

^a^ count per 100,000

**Figure S1.** Cumulative risk curve for breast cancer.

**Table S3.** Decreases in breast density with age.

| % of women with BI-RADS d at age 50 y | BI-RADS density according to age | | |
| --- | --- | --- | --- |
|  | 50-59 y | 60-69 y | ≥ 70 y |
| 23% | d | d | d |
| 8% | d | d | c |
| 54% | d | c | c |
| 15% | d | c | b |

**Validation**

We externally validated our model by using observed data from the DENSE trial (3,4). The median baseline age of women in the mammography-only group and MRI participants was 54 years (IQR: 51-61) and 54 years (IQR: 51-59), respectively (3). For comparability, the model was externally validated by simulating two screening rounds at ages 54 and 56.

**Table S4.** Validation of interval cancers, screen-detected cancers and false positives from the simulation.

|  | Observed data (95% CI) ^a^ | Simulation results (95% CI) ^b^ |
| --- | --- | --- |
| **Mammography only group** |  |  |
| Number of interval cancers in the first round |  |  |
| T1 (≤2 cm) | 75 (59-94) | 127 (119-134) |
| T2+ (>2cm） | 77 (61-96) | 39 (28-52) |
| Total | 152 (129-178) | 165 (147-185) |
| Number of screen-detected cancers in the second round |  |  |
| T1 (≤2 cm) | 80 (63-100) | 113 (105-144) |
| T2+ (>2cm） | 32 (22-45) | 41 (37-52) |
| Total | 112 (92-135) | 154 (129-181) |
| **MRI participants** |  |  |
| Number of interval cancer in the first round by MRI |  |  |
| T1 (≤2 cm) | 3 (1-9) | 8 (5-10) |
| T2+ (>2cm） | 1 (0-6) | 0 (0-1) |
| Total | 4 (1-10) | 8 (5-11) |
| Number of screen-detected cancer in the first round by MRI |  |  |
| T1 (≤2 cm) | 57 (43-74) | 33 (25-44) |
| T2+ (>2cm） | 7 (3-14) | 1 (1-2) |
| Total | 64 (49-82) | 34 (26-46) |
| Number of false positives in the first round by MRI | 390 (352-431) | 380 (341-418) |
| Number of screen-detected cancer in the second round by mammography |  |  |
| T1 (≤2 cm) | 3 (1-9) | 9 (8-11) |
| T2+ (>2cm） | 2 (0-7) | 1 (0-1) |
| Total | 5 (2-12) | 10 (8-11) |
| Number of screen-detected cancer in the second round by MRI |  |  |
| T1 (≤2 cm) | 14 (8-23) | 24 (20-28) |
| T2+ (>2cm） | 0 (0-4) | 0 (0-0) |
| Total | 14 (8-23) | 24 (20-28) |
| Number of false positives in the second round by MRI | 96 (78-117) | 274 (247-300) |

^a^ Data from Bakker et al. (3) and Geuzinge et al. (4).

^b^ Screened at ages 54 and 56 (3).

**Figure S2.** Effect of varying CEM sensitivity on the ICER of the CEM alone screening strategy. Abbreviations: ICER = Incremental cost-effectiveness ratio.

**Table S5.** Scenario analysis using an alternative Dutch estimate of the CEM examination cost.

| Scenario | CEM cost/screen | ICER (€K/LYG) ^a^ |
| --- | --- | --- |
| Base-case | €162 | 13.4 |
| Published Dutch estimate | €111 ^b^ | 7.8 |

Abbreviations: LYG = life years gained; ICER = incremental cost-effectiveness ratio; CEM = contrast-enhanced mammography.

^a^ CEM alone screening strategy vs the reference.

^b^ Data from the recently published Dutch estimate reported by Neeter et al (5).

References

1.	IKNL. Cijfers over kanker. [Internet]. Available from: http://www.cijfersoverkanker.nl/

2.	Schousboe JT, Kerlikowske K, Loh A, Cummings SR. Personalizing mammography by breast density and other risk factors for breast cancer: analysis of health benefits and cost-effectiveness. Ann Intern Med. 2011 Jul 5;155(1):10–20. doi:10.7326/0003-4819-155-1-201107050-00003 PubMed PMID: 21727289; PubMed Central PMCID: PMC3759993.

3.	Bakker MF, de Lange SV, Pijnappel RM, Mann RM, Peeters PHM, Monninkhof EM, et al. Supplemental MRI Screening for Women with Extremely Dense Breast Tissue. N Engl J Med. 2019 Nov 28;381(22):2091–102. doi:10.1056/NEJMoa1903986 PubMed PMID: 31774954.

4.	Geuzinge HA, Bakker MF, Heijnsdijk EAM, van Ravesteyn NT, Veldhuis WB, Pijnappel RM, et al. Cost-Effectiveness of Magnetic Resonance Imaging Screening for Women With Extremely Dense Breast Tissue. J Natl Cancer Inst. 2021 Nov 2;113(11):1476–83. doi:10.1093/jnci/djab119 PubMed PMID: 34585249; PubMed Central PMCID: PMC8562952.

5.	Neeter LMFH, Lobbes MBI, Duvivier KM, Raat HPJF, Smidt ML, Wildberger JE, et al. Cost-effectiveness of contrast-enhanced mammography compared to conventional imaging in the work-up after screening (RACER trial). Eur Radiol. 2026 May;36(5):3396–405. doi:10.1007/s00330-025-12195-8 PubMed PMID: 41441997.
